# Elbow stiffness due to malunited capitellum fracture: A case report and the role of 3D printing in surgical management

**DOI:** 10.1016/j.ijscr.2024.110398

**Published:** 2024-09-30

**Authors:** Erica Kholinne, Karina Sylvana Gani, Mitchel, William, Erick Wonggokusuma, Ameria Pribadi

**Affiliations:** a Gatam Institute, Eka Hospital, Indonesia; b Faculty of Medicine, Universitas Trisakti, Jakarta, Indonesia

**Keywords:** Malunion capitellum fracture, Stiff elbow, Post-traumatic, Case report, Lateral-column approach

## Abstract

**Introduction:**

Elbow stiffness is an uncommon condition that significantly impacting a patient's daily activities. Trauma is the most frequent cause of elbow stiffness. However, capitellum fractures are rare, accounting for approximately 1 % of elbow fractures. They are often misdiagnosed due to nonspecific symptoms and the complex anatomy of the elbow joint.

**Case presentation:**

We report the case of a 54-year-old female who presented with left elbow stiffness eight months after a traumatic incident. On physical examination, her left elbow extension was +10°, and flexion was restricted to 65°, with no limitation in pronation or supination. Imaging studies revealed a malunited capitellum with osteophytes at the posterolateral site of the olecranon. A 3D-printed model of her elbow was created based on a CT scan to aid surgical planning. She underwent capsulectomy and osteotomy and was stabilized with two bioabsorbable P(L/DL)LA pins. Six months postoperatively, the patient's elbow range of motion was fully restored, and no complications were observed.

**Clinical discussion:**

Elbow stiffness resulting from the malunion of a capitellum fracture typically necessitates surgical intervention to restore functional movement in the elbow.

**Conclusion:**

Capitellum fractures are uncommon and frequently underdiagnosed, leading to complications such as elbow stiffness and reduced functionality. Early detection is crucial, as delayed diagnosis can result in complex management due to malunion. 3D printing from CT scans helps surgeons accurately evaluate malunions and plan precise surgical interventions.

## Highlights

• Early recognition of capitellum fractures is essential to prevent future complications
• Capitellum fracture is rare and sometimes is underdiagnose
• Malunion from an undiagnosed capitellum fracture can cause elbow stiffness
• 3D printing from CT scans helps surgeons evaluate malunion and surgical plans accurately

## 1 Introduction

Post-traumatic elbow stiffness is relatively rare, occurring in approximately 5 % of cases [1]. Capitellum fractures are rare, accounting for around 1 % of all elbow fractures and 6 % of all distal humerus fractures [2]. Despite their rare occurrence, detecting capitellum fractures can be challenging due to their small size, articular nature, and tendency to displace, which can obstruct elbow motion [3]. Morrey et al. Reported that the functional arc of elbow motion required for daily activities is 100° for flexion and extension (ranging from 30 to 130°) and 50° in either direction for pronation and supination [4]. Elbow stiffness is a reduction in extension more significant than 30° or a flexion less than 120 degrees [5]. This condition can make it difficult for patients to perform their daily activities. The most common cause of elbow stiffness is trauma. Neglected elbow trauma can result in elbow stiffness and usually fails to address the underlying elbow pathology [6]. Our case has been reported in line with the SCARE criteria [7].

## 2 Case presentation

### 2.1 Preoperative evaluation

A 54-year-old female patient was admitted to an orthopedic outpatient clinic with a restricted range of motion in her left elbow following a fall from a motor vehicle accident eight months ago. The patient initially sought treatment at a local hospital and was diagnosed with a sprained elbow. Despite undergoing a regimen of home exercises and anti-inflammatory medication, there was no significant improvement in her range of motion. Subsequently, the patient sought manual therapy at a traditional medicine institution, which also did not yield positive results. Consequently, she is experiencing difficulty with daily activities, such as personal hygiene.

**Fig. 1:**
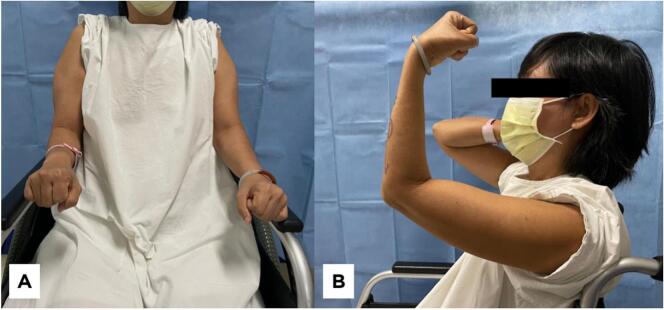
Range of motion of the left elbow. Extension +10^o^ and flexion is limited to 65^o^.

Upon physical examination, it was observed that the range of motion (ROM) in the patient's left elbow was limited to +10 for extension to 65° in flexion, with no restriction in pronation and supination and no observed deficit in rotational arc (Fig. 1). The collateral ligaments were stable, and no ulnar nerve deficit was detected. There were no deficits in motor strength, and sensory function was intact. Additionally, there was no evidence of muscle atrophy. (See Table 1.)

**Fig. 2:**
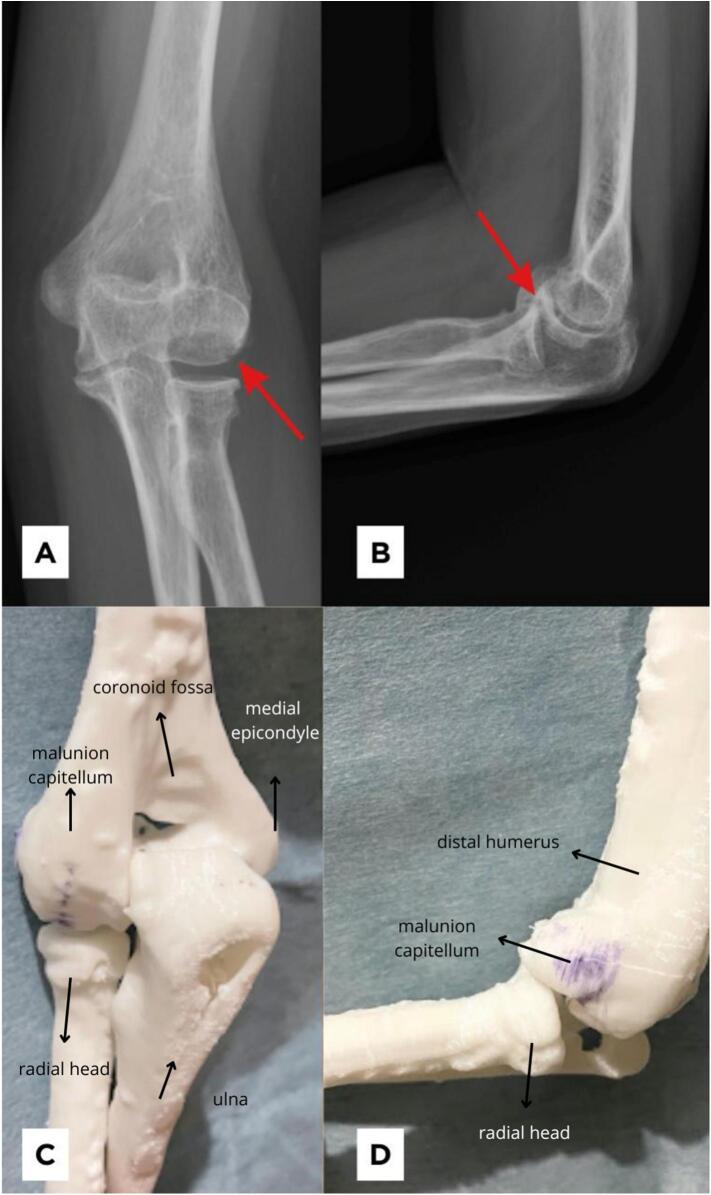
Imaging studies of left elbow. Plain radiograph of the elbow in (A) AP position and (B) lateral position, showing the characteristic double-arc sign (arrow) revealing a malunited (coronal sheer) in humeral capitellum. (C&D) A 3D-printed model of the patient's left elbow was made based on a CT scan showing a malunion of humeral capitellum.

**Table 1:** The Timeline of Events.

Timeline	
Eight-months preoperative	Motor vehicle accident causing restriction in her left elbow; patient undergoes a regimen of home exercises, anti-inflammatory medication, and traditional medicine
Preoperative	Left elbow ROM: +10° for extension to 65° degrees in flexion X-ray and CT scan: Malunited capitellum resulting from a suspected previous coronal shear fracture, impeding elbow flexion 3D-printing model is made
Operative	Capsulectomy, debridement, and osteotomy were performed
Two weeks postoperative	Active elbow flexion extension and wears a night splint in the following 2 to 6 weeks.
Two-months postoperative	Left elbow ROM: 0° for extension to 120° degrees in flexion
Six-months postoperative	Left elbow ROM: 0° for extension to 150° degrees in flexion
One-year postoperative	Plain radiograph: no osteolytic reaction around the insertion of the pin The patient expressed high satisfaction with the postoperative outcomes. She reported no pain during daily activities and experienced no side effects. Quickdash score 56.8 to 2.3 VAS 3 to 0

ROM, range of motion; CT, computed tomography; DASH, Disabilities of the Arm, Shoulder and Hand; VAS, visual analogue scale.

Imaging studies, including plain X-rays and a CT scan (video 1), revealed a malunited capitellum resulting from a suspected previous coronal shear fracture, impeding elbow flexion. Furthermore, osteophytes were noted at the posterolateral site of the olecranon, potentially limiting elbow extension. No signs of heterotopic ossification were identified. After a thorough discussion with the patient, preoperative planning involved utilizing a 3D-printed model of her elbow created from the CT scan (Fig. 2). It was decided that a corrective osteotomy, preceded by the removal of the posterolateral olecranon osteophyte, would be performed.

### 2.2 Surgical procedure

**Fig. 3:**
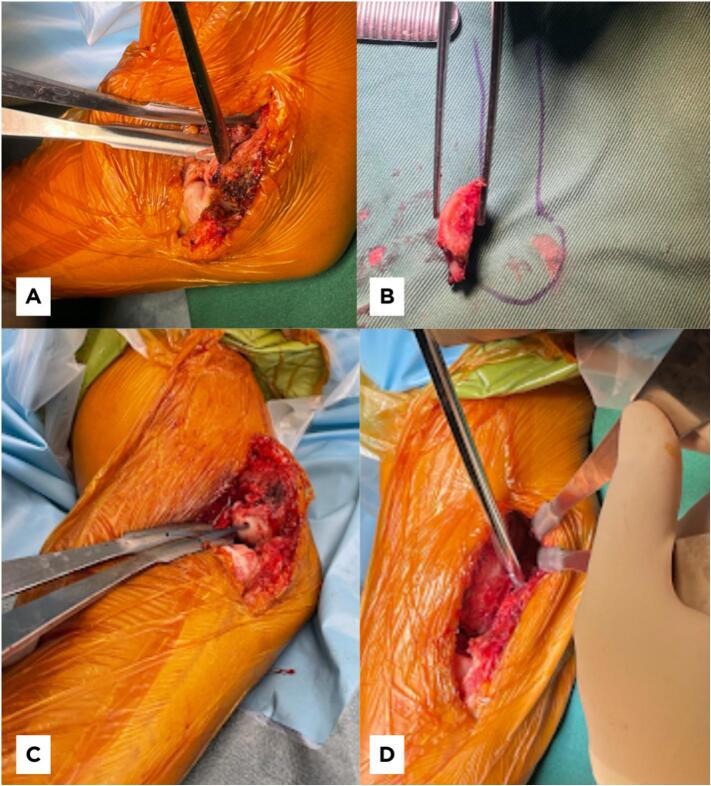
Intraoperative images. (A &B) The malunited capitellum was osteotomized from the distal humerus. (C) The capitellum was fixed with two bioabsorbable pins. (D) The osteophytes were excised from the posterolateral margin of the olecranon with a motorized burr.

During the surgical procedure, the patient was placed under general anesthesia in a prone position, with a tourniquet set at 250 mmhg. A lateral column approach was employed, revealing the presence of a dense fibrous capsule at the anterior part of the elbow joint. Capsulectomy was performed, along with the debridement of fibrous tissue. The malunited capitellum was identified, and a corrective osteotomy was performed based on the preoperative plan derived from the 3D-printed model. Following meticulous debridement and scar tissue removal from the distal humerus bony bed, the capitellum was realigned and secured with two bioabsorbable P(L/DL)LA pins. Given the patient's reluctance to undergo a second surgery, non-hardware fixation was chosen. Subsequently, the posterolateral olecranon spur was removed, enabling a full range of motion return (Fig. 3). The humeral attachment of the lateral collateral ligament complex was reflected and tagged from its insertion to facilitate the removal of the posterolateral olecranon. Following the full range of motion restoration, the lateral collateral ligament complex was repaired with a suture anchor.

### 2.3 Postoperative care

Soft compressive dressing and a posterior splint were applied to the patient. The surgeon performed a Neurologic examination after the patient recovered from anesthesia. Active elbow flexion-extension was started after two weeks postoperatively as the splint was removed and the wound healed. With assistance from the contralateral hand, the patient increases the ROM in extension and flexion and wears a night splint in the following 2 to 6 weeks. The patient was given oral celecoxib (200 mg, twice daily) for two weeks to minimize the possibility of heterotopic ossification.

**Fig. 4:**
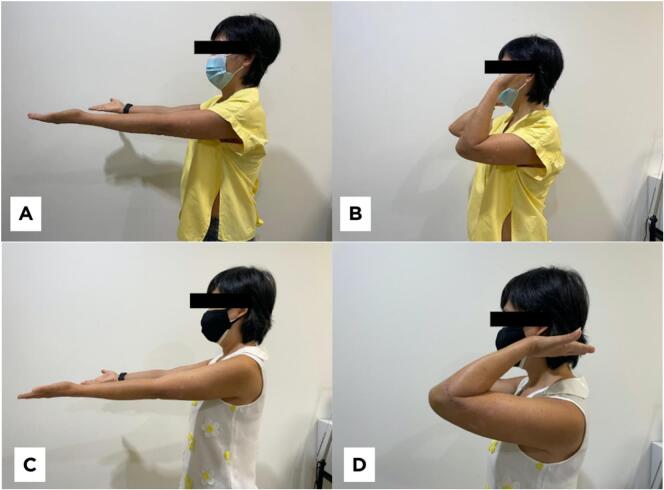
Post-operative follow-up. (A&B) Two months post-operative shows a motion arc of 120° (extension - flexion: 0° - 120°). (C&D) Six months post-operative shows motion arc of 150° (extension - flexion: 0° - 150°).

**Fig. 5:**
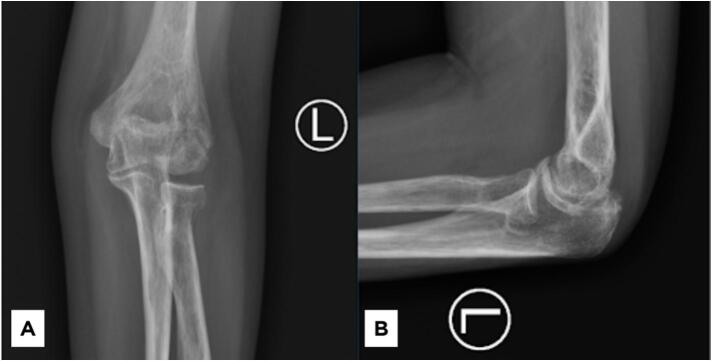
A one-year post-operative X-ray in the AP and lateral positions shows no osteolytic reaction around the insertion of the pin.

Two months after surgery, her elbow movement displayed improvement with a flexion of approximately 100°. Six months after surgery, the patient visited the surgical outpatient clinic for a post-operative mobility assessment. Her elbow range of motion was restored upon physical examination, and no avascular necrosis was found (Fig. 4). At the one-year follow-up, plain radiologic imaging revealed no osteolytic lesions around the pin insertion site (Fig. 5). The patient expressed high satisfaction with the postoperative outcomes with quickdash score ranged from 56.8 to 2.3. She reported no pain (Visual analog scale ranged from 3 to 0) during daily activities and experienced no side effects or complications from the time of surgery through the one-year follow-up.

## 3 Discussion

Isolated capitellum fracture is a rare injury pattern that only presents for an average of 1 % of all elbow fracture cases and may result in malunion of the fracture fragment if misdiagnosed [8]. Delayed treatment has been described by Sing et al., who presented ten weeks after injury [9]. In the current report, our patient went to the local hospital emergency department immediately following the injury; the fracture remained undetected, resulting in a malunion of the fracture fragment, which has also been reported by Elmaraghy et al. If a fracture is left undetected, it could lead to severe limitation of range of motion due to malunion [10]. These injuries may be complex to detect on physical examination and can remain undetected on imaging if they are not suspected.

Early recognition of capitellum fractures is essential for management to prevent future complications. Physicians should explore the medial aspect of the elbow and look for irregularities of the trochlear surface on radiographs, including unexpected double shadow or contour. This is particularly important in women with a history of traumatic elbow injuries, as they tend to have a higher carrying angle and may also be affected by postmenopausal osteoporosis [11]. Additionally, oblique views, comparison views of the opposite side, and CT examinations can all help detect these injuries.

In recent years, several studies highlight the importance and significant benefits that can be derived from utilizing 3D printing models. 3D printing is an additive manufacturing technique used to produce various products with complex geometries and structures derived from three-dimensional models. This technique provides valuable production capabilities for medical components, including patient-specific implants, prosthetics, and tissue scaffolds [[12], [13], [14], [15]]. 3D printing technology has matured, allowing orthopedic surgeons to use preoperative CT data to print visual models of fractures, thereby enabling more comprehensive preoperative diagnosis, simulation, and surgical planning [16]. The use of 3D printing technology and customization in orthopedics has become a reality [16]. Yoshii et al. Employed three-dimensional preoperative planning to treat distal radius fractures. The reproducibility of fracture reduction was relatively high, and the reproducibility of implant selection was outstanding [17]. Three-dimensional digital planning helps visualize the reduction process and choose appropriate implants to address distal radius fractures. Furthermore, Yoshii et al. Demonstrated the significant benefits of preoperatively determining the plate and screw positions and types for distal radius fractures through 3D printing and image fusion systems [18]. Grunert et al. Also use 3D-printing model in stiff elbow case. They explained Haptic 3D printed models help the surgeon to identified the difficulties so the surgeon can anticipated intraoperatively and precise resection planning also can be achieved [19]. This align with our study by using the 3D-printing model based on CT scan to help the surgeon to have a better vision of the anatomical shape and a more realistic depiction of the fracture. Other study also found the advantages of using 3D-printing model compared to traditional surgical planning using two-dimensional imaging; preoperative planning with fractured 3D models has demonstrated shorter surgical times, reduced blood loss, and improved elbow function in outcomes [20].

Post-traumatic elbow stiffness is uncommon, only appears in 5 % of cases, and is mainly found in young adults [1]. It causes significant functional impairment because it limits the patient's elbow range of motion to perform functional activities [21]. Elbow stiffness is defined as having a flexion-extension arc of less than 100° and/or a flexion contracture greater than 30°. The reasons why the elbow is susceptible to contracture remain unclear, and it is also not well understood which patient characteristics are associated with post-traumatic elbow stiffness [21].

Morrey categorized joint stiffness's etiology and anatomical location into intrinsic, extrinsic, and mixed types. Intrinsic stiffness was associated with joint conditions, while extrinsic stiffness was confined to soft tissues or extra-articular processes. In mixed type, intrinsic pathology evolves into extrinsic contracture [22]. The current study reflected post-traumatic stiffness due to mixed type because of malunion as an intrinsic factor and capsular contracture as an irrelevant factor. An intra-articular distal humeral fracture is reported to be more prone to stiffness than an extra-articular fracture, which is reflected in the current case [23].

In this case, we use a lateral-column approach. The original lateral-column approach described by Mansat and Morrey effectively improves the elbow's flexion-extension arc, with an average increase of 45° and a complication rate of 11 %. The lateral column approach allows the surgeon to address the anterior and posterior aspects of the capsule, which is attributed to the cause of stiffness in the current case. Various methods for treating capitellum fracture can be done, such as K-wires, headless compression screws, and absorbable implants. Absorbable implants offer several advantages: no need for removal surgery, osteoconductive properties that promote bone growth, gradual load transfer to the bone as the implant absorbs, and easier recovery without the mechanical issues of metal implants. [24].

Kruse et al. Reported on 36 patients with stiff elbows who underwent a lateral-column approach combined with posterior mini-open access, with an average follow-up of 38 months. All patients showed improvements in their range of motion. For those with post-traumatic contractures, elbow flexion improved from 99° to 128° at the final follow-up, while those with degenerative elbow contractures improved from 98° to 126°. Similarly, extension improved from an average of 52° to 19° in the post-traumatic group and from 41° to 17° in the degenerative stiffness group. The average gain in flexion extension across both groups was 57° [25]. Ring et al. Conducted a study involving 46 participants with post-traumatic stiff elbows who underwent capsulotomy. The results showed an improvement in elbow arc to nearly 100°. Additional surgery is needed in 14 patients with persistent elbow contracture, ulnar neuropathy, or both, and giving additional improvement in ROM (±26°) [26].

Jeevannavar et al. Reported a case of 20 years old woman with a malunited capitellum fracture who underwent corrective osteotomy and internal fixation using a Herbert screw, giving a good result with a functional elbow and absence of pain [27]. The same result was reported by Bilic et al. In their study of 11-year-old girls with malunited left capitellum fracture who underwent osteotomy reconstruction using absorbable implants, giving medial and lateral stability to her elbow. She also fully uses her upper extremity with no complaint of pain [28].

Although the utilization of 3D printing models is currently limited, it demonstrates considerable potential for broader adoption in the future. These technologies are expected to improve, allowing for more realistic analyses of complex scenarios even on lower-spec devices [[29], [30], [31], [32]]. We believe that 3D printing models could prove invaluable for surgeons in visualizing complex anatomy and selecting the most appropriate treatment or surgical strategy for post-traumatic stiff elbow. However, further research with larger sample sizes is necessary to validate these benefits.

## 4 Conclusion

Capitellum fractures are a rare injury and are frequently underdiagnosed or not initially considered, leading to diagnostic challenges in the early stages. However, prompt identification of capitellum fractures is vital because delayed diagnosis or neglected fractures can lead to elbow stiffness, significantly impairing function, and daily activities. Management becomes increasingly complex once elbow stiffness or contracture develops, partly due to malunion in neglected capitellum fractures. Hence, 3D printing using CT scans can help surgeons analyze the precise shape of malunions, enabling detailed and carefully planned surgical interventions. In diagnosing capitellum fractures, 3D CT scans are crucial for overcoming the limitations of traditional imaging methods. They provide enhanced visualization, allowing surgeons to analyze the precise shape of the malunion. This enables detailed and carefully planned surgical interventions, ensuring patients receive the most appropriate and effective treatment.

The following is the supplementary data related to this article.

Video 1 CT scan left elbow Video 1

## Ethical approval

This is a case report; appropriate informed consent has been obtained from the patient. Ethical approval for the case report has been exempted by the ethical committee of the Faculty of Medicine, University of Trisakti on July, 16th, 2024.

## Funding

This research received no specific grant from funding agencies in the public, commercial, or not-for-profit sectors.

## Authorship contribution

Erica Kholinne: Conceptualization, Data curation, Investigation, Resources. Erick Wonggokusuma, Ameria Pribadi, William: Formal analysis, Writing - review & editing. Karina Sylvana Gani, Mitchel: Methodology, Project administration. Erica Kholinne, Erick Wonggokusuma: Supervision. Ameria Pribadi: Validation. William: Visualization. Erica Kholinne, Karina Sylvana Gani, Mitchel: Roles/Writing - original draft. All authors have read and approved the final manuscript.

## Guarantor

Erica Kholinne.

## Registration of research studies

N/A

## Consent

Written informed consent was obtained from the patient for publication and any accompanying images. A copy of the written consent is available for review by the Editor-in-Chief of this journal on request.

## Conflict of interest

None.
